# Risk factors for type 2 diabetes mellitus: An exposure-wide umbrella review of meta-analyses

**DOI:** 10.1371/journal.pone.0194127

**Published:** 2018-03-20

**Authors:** Vanesa Bellou, Lazaros Belbasis, Ioanna Tzoulaki, Evangelos Evangelou

**Affiliations:** 1 Department of Hygiene and Epidemiology, University of Ioannina Medical School, Ioannina, Greece; 2 Department of Epidemiology and Biostatistics, School of Public Health, Imperial College London, London, United Kingdom; 3 MRC-PHE Centre for Environment and Health, School of Public Health, Imperial College London, London, United Kingdom; University of Hawai'i at Manoa College of Tropical Agriculture and Human Resources, UNITED STATES

## Abstract

**Background:**

Type 2 diabetes mellitus (T2DM) is a global epidemic associated with increased health expenditure, and low quality of life. Many non-genetic risk factors have been suggested, but their overall epidemiological credibility has not been assessed.

**Methods:**

We searched PubMed to capture all meta-analyses and Mendelian randomization studies for risk factors of T2DM. For each association, we estimated the summary effect size, its 95% confidence and prediction interval, and the I^2^ metric. We examined the presence of small-study effects and excess significance bias. We assessed the epidemiological credibility through a set of predefined criteria.

**Results:**

We captured 86 eligible papers (142 associations) covering a wide range of biomarkers, medical conditions, and dietary, lifestyle, environmental and psychosocial factors. Adiposity, low hip circumference, serum biomarkers (increased level of alanine aminotransferase, gamma-glutamyl transferase, uric acid and C-reactive protein, and decreased level of adiponectin and vitamin D), an unhealthy dietary pattern (increased consumption of processed meat and sugar-sweetened beverages, decreased intake of whole grains, coffee and heme iron, and low adherence to a healthy dietary pattern), low level of education and conscientiousness, decreased physical activity, high sedentary time and duration of television watching, low alcohol drinking, smoking, air pollution, and some medical conditions (high systolic blood pressure, late menarche age, gestational diabetes, metabolic syndrome, preterm birth) presented robust evidence for increased risk of T2DM.

**Conclusions:**

A healthy lifestyle pattern could lead to decreased risk for T2DM. Future randomized clinical trials should focus on identifying efficient strategies to modify harmful daily habits and predisposing dietary patterns.

## Background

Type 2 diabetes mellitus (T2DM) ranks highly on the international health agenda as a global pandemic and as a threat to human health and global economies. The number of people with T2DM worldwide has more than doubled during the past 20 years [1]. According to the International Diabetes Federation, 415 million people are living with T2DM in 2015, and by 2040 the number will be almost 642 million [2]. These estimates correspond to a global prevalence of 8.8% (95% confidence interval, 7.2–11.4%) in 2015, and a projected global prevalence of 10.4% (95% confidence interval, 8.5–13.5%) in 2040 [2]. Epidemiological data predict an inexorable and unsustainable increase in global health expenditure attributable to T2DM, so disease prevention should be given high priority.

T2DM results from an interaction between genetic and environmental factors [3]. Genes and the environment together are important determinants of insulin resistance and β-cell dysfunction [4]. Because changes in the gene pool cannot account for the rapid increase in prevalence of T2DM in recent decades, environmental changes are essential to the understanding of the epidemic.

Systematic reviews and meta-analyses of observational studies have indicated numerous risk factors for T2DM. However, the epidemiological credibility of these associations has not been appraised across the field. In the present work, we performed an umbrella review of the evidence across existing systematic reviews and meta-analyses of observational studies that examine any non-genetic risk factor for T2DM. We primarily aim to provide an overview of the range and validity of the reported associations of diverse environmental risk factors and biomarkers with T2DM. Furthermore, we assessed whether there is evidence for diverse biases and which of the previously studied associations have robust evidence.

## Materials and methods

### Search strategy and eligibility criteria

We conducted an umbrella review, i.e. a comprehensive and systematic collection and evaluation of systematic reviews and meta-analyses performed on a specific research topic using previously described and applied methodology [5–12].

We systematically searched PubMed from inception until February 10, 2016 to identify systematic reviews and meta-analyses of observational studies examining associations of non-genetic risk factors with T2DM. We used the following search strategy: diabetes AND (“systematic review” OR meta-analysis). Two independent investigators (VB, LB) retrieved and abstracted the full text of potentially eligible articles. We excluded meta-analyses that investigated the association between genetic polymorphisms and risk for T2DM; that included less than 3 component studies; that included studies with overlapping populations; that included studies using different units of comparison of the same exposure without transforming the effect estimates appropriately. We further excluded meta-analyses performing comparison between drug agents and subsequent risk for developing T2DM in population at high risk. When an association was covered by more than one meta-analyses, we kept the meta-analysis including the largest number of component studies and adequately presenting the study-specific effect estimates and sample sizes of component studies. We did not apply any language restrictions in our search strategy.

Finally, in order to assess the causality of the associations between the reported risk factors and T2DM, we conducted an additional systematic search on PubMed to capture mendelian randomization (MR) studies for T2DM. This search algorithm used the keywords: “mendelian randomization” OR “mendelian randomisation”. MR studies were eligible if they studied T2DM and examined the potentially causal effect of a risk factor that was also included in our umbrella review. We excluded studies focused on impaired glucose tolerance, impaired fasting glucose or insulin resistance as outcomes.

### Data extraction

Two independent investigators (VB, LB) extracted the data, and in case of discrepancies consensus was reached. From each eligible article, we abstracted information on the first author, journal and year of publication, the examined risk factors and the number of studies considered. We also extracted the study-specific risk estimates (i.e. risk ratio, odds ratio, hazard ratio, standardized mean difference) along with their 95% confidence interval (CI) and the number of cases and controls in each study. If a meta-analysis included multiple effect estimates from the same observational study using the same control group, we included only the effect estimate that corresponded to the largest sample size.

From each eligible MR study, we extracted the first author and year of publication, the definition of outcome, the risk factor considered, the level of comparison for exposure, the genetic instrument used, the applied statistical approach, the sample size, the causal odds ratio and its 95% CI, the P-value for the association, and whether the authors claimed that a causal relationship exists. If an MR study used a genetic instrument based on a single variant and a genetic instrument based on polygenic risk score (PRS), we extracted the information from the PRS, as this approach is more powerful.

### Statistical analysis

For each meta-analysis, we estimated the summary effect size and its 95% CI using both fixed-effect and random-effects models. [13,14] We also estimated the 95% prediction interval (PI), which accounts for the between-study heterogeneity and evaluates the uncertainty for the effect that would be expected in a new study addressing that same association. [15,16]

Between-study heterogeneity was quantified using the I^2^ metric. I^2^ ranges between 0% and 100% and quantifies the variability in effect estimates that is due to heterogeneity rather than sampling error. [17] Values exceeding 50% or 75% are considered to represent large or very large heterogeneity, respectively. This step is necessary to ensure that all results from each meta-analysis are available to assess the epidemiological credibility of the associations.

We assessed small-study effects using the Egger’s regression asymmetry test. [18,19] A P <0.10 combined with a more conservative effect in the largest study than in random-effects meta-analysis was judged to provide adequate evidence for small-study effects. We further applied the excess statistical significance test, which evaluates whether there is a relative excess of formally significant findings in the published literature due to any reason. [20] We used the effect size of the largest study (smallest standard error) in each meta-analysis to calculate the power of each study using a non-central *t* distribution. [21,22] Excess statistical significance was claimed at two-sided P <0.10. [21] In two meta-analyses (glycemic load as dichotomous exposure, and breastfeeding), the excess significance test was not performed, because the sample size was not reported in some of the component studies.

### Assessment of epidemiological credibility

We identified associations that had the strongest evidence and no signals of large heterogeneity or bias. We considered as *convincing* the associations that fulfilled all the following criteria: statistical significance per random-effects model at P <10^−6^; based on >1,000 cases; without large between-study heterogeneity (I^2^<50%); 95% PI excluding the null value; and no evidence of small-study effects and excess significance bias. Associations with >1,000 cases, P <10^−6^ and largest study presenting a statistically significant effect were graded as *highly suggestive*. The associations supported by >1,000 cases and a significant effect at P <10^−3^ were considered as *suggestive*. The remaining nominally significant associations (P <0.05) were considered as having *weak evidence*.

For associations with convincing and highly suggestive evidence, we performed a sensitivity analysis limited to prospective cohort studies and nested case-control studies, and we examined whether there was a change in the level of epidemiological credibility. Also, we compared the findings from the meta-analyses of observational studies with the findings from MR studies.

The statistical analysis and the power calculations were done with STATA version 12.0 and RStudio version 1.0.44.

## Results

### Eligible studies

**Fig 1:**
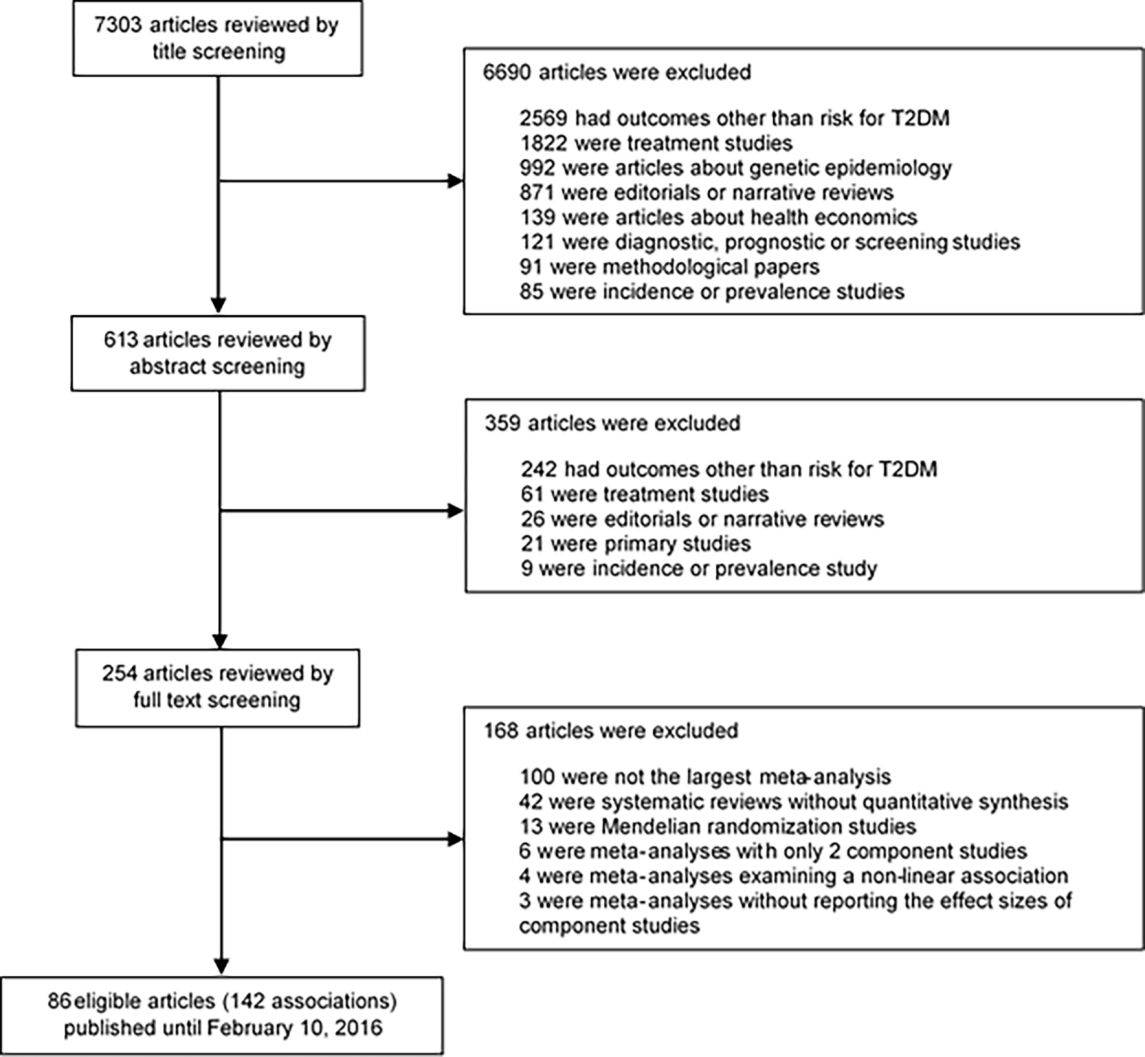
Flow chart of literature search.

Our literature search yielded 7,303 papers, of which 86 papers met our inclusion criteria (Fig 1). Fourteen papers, including 16 associations (i.e., sedentary time, breakfast skipping, psoriasis, psoriatic arthritis, breastfeeding, adverse childhood experience, height, hip circumference, serum osteocalcin, spousal diabetes, osteoarthritis, polycystic ovary syndrome, schizophrenia, major depressive disorder, and bipolar disorder), combined cross-sectional studies with either cohort studies or case-control studies in their analysis.

The 86 eligible papers examined 109 unique risk factors and 142 associations related to risk for developing T2DM. These associations covered a wide range of exposures: biomarkers (n = 25 associations), dietary factors (n = 53 associations), lifestyle factors and environmental exposures (n = 22 associations), medical history (n = 16 associations), metabolic factors and anthropometric traits (n = 15 associations), and psychosocial factors (n = 11 associations). The median number of cases per meta-analysis was 8,825 (IQR, 2,892–17,782), and the median number of datasets was 10 (IQR, 6–14). Only 7 meta-analyses included less than 1,000 T2DM cases.

### Statistically significant associations, heterogeneity and biases

**Fig 2:**
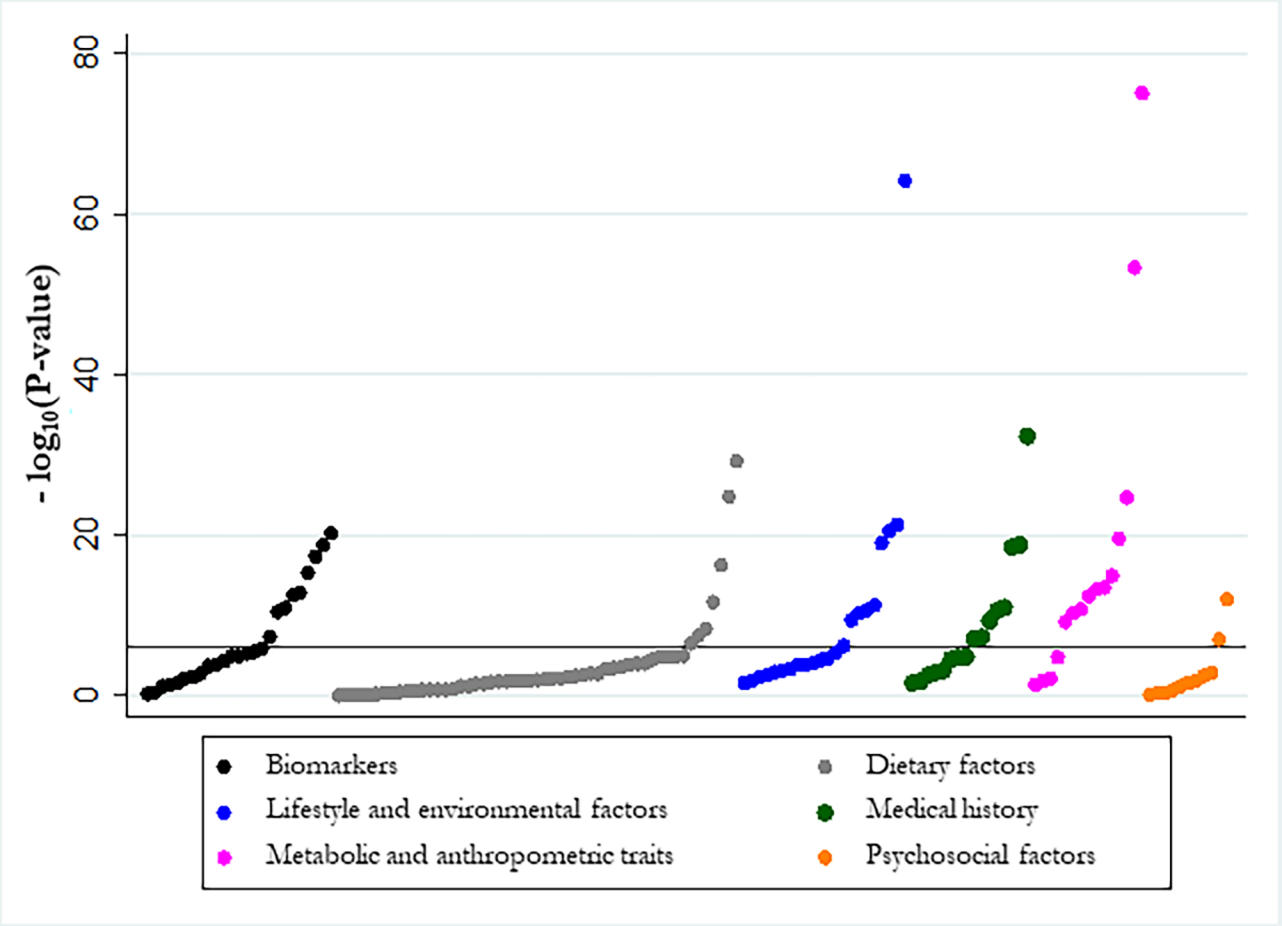
Manhattan plot for 142 associations between risk factors and T2DM. The horizontal line corresponds to the significance threshold of P <10^−6^.

One hundred and sixteen of 142 associations (82%) presented a statistically significant effect at P <0.05 under the random-effects model, whereas 46 associations had a statistically significant effect at P <10^−6^ (Table 1). Fig 2 displays the distribution of the P-values in each category of associations. Only 33 of 142 associations (23%) had a 95% PI that excluded the null value and 26 of these also had a P <10^−6^.

**Table 1:** Characteristics of 142 associations between non-genetic risk factors and type 2 diabetes mellitus.

Reference	Risk factor	Level of comparison	Number of cases/controls	Number of datasets	Effect size metric	Random-effects summary effect size (95% CI)	P random	95% prediction interval	I^2^	Small-study effects/Excess significance bias	Grading
*Biomarkers*											
Aune, 2015 [57]	Resting heart rate	Per 10 bpm increase	6217/106,601	9	RR	1.20 (1.07–1.35)	1.74 × 10^−3^	0.80–1.79	93.4	No/Yes	Weak
Chen, 2014 [58]	Serum leptin	Per 1 log ng/ml increase	4084/22,367	17	RR	1.13 (1.01–1.27)	0.038	0.74–1.73	76	Yes/Yes	Weak
Emdin, 2015 [39]	Systolic blood pressure	Per 20 mmHg increase	204,803/4,212,999	40	RR	1.75 (1.56–1.97)	6.15 × 10^−21^	0.97–3.16	85.7	No/No	Highly suggestive
Fraser, 2009 [59]	Serum ALT	Per 1 log unit increase	2009/32,292	14	HR	1.85 (1.57–2.18)	2.85 × 10^−13^	1.31–2.61	19.2	No/No	Convincing
Fraser, 2009 [59]	Serum ALT	Highest vs. lowest category	1087/22,729	10	HR	2.07 (1.54–2.79)	1.52 × 10^−6^	1.07–4.02	27.3	No/No	Suggestive
Fraser, 2009 [59]	Serum γGT	Highest vs. lowest category	1352/20,955	10	HR	3.07 (2.22–4.23)	1.02 × 10^−11^	1.60–5.86	19.9	Yes/No	Highly suggestive
Fraser, 2009 [59]	Serum γGT	Per 1 log unit increase	2742/60,173	18	HR	1.92 (1.66–2.21)	1.58 × 10^−19^	1.20–3.07	54.8	No/No	Highly suggestive
Jia, 2013 [60]	Serum uric acid	Highest vs. lowest category	5115/43,693	11	RR	1.60 (1.44–1.78)	4.60 × 10^−18^	1.39–1.85	3.4	No/No	Convincing
Kodama, 2009 [61]	Serum uric acid	Per 1 mg/dl increase	3305/39,529	14	RR	1.17 (1.09–1.25)	1.15 × 10^−5^	0.92–1.48	74.8	Yes/Yes	Suggestive
Kunutsor, 2013 [25]	Serum ferritin	Highest vs. lowest category	3391/22,948	9	RR	1.73 (1.35–2.22)	1.23 × 10^−5^	0.84–3.56	58.2	No/No	Suggestive
Kunutsor, 2013	Serum AST	Highest vs. lowest category	5985/79,958	11	RR	1.26 (1.11–1.42)	1.98 × 10^−4^	0.89–1.78	56.4	Yes/Yes	Suggestive
Kunutsor, 2013	Serum AST	Per 1 SD increase	1828/20,290	7	RR	1.13 (1.02–1.25)	0.021	0.85–1.49	52.5	No/Yes	Weak
Kunutsor, 2015 [62]	Serum osteocalcin	Highest vs. lowest category	1673/6963	9	RR	0.43 (0.29–0.65)	5.56 × 10^−5^	0.12–1.52	87.8	Yes/Yes	Suggestive
Lee, 2009 [63]	Serum CRP	Highest vs. lowest category	3920/24,914	16	RR	1.79 (1.51–2.13)	3.30 × 10^−11^	1.03–3.11	53.4	No/No	Highly suggestive
Li, 2009 [64]	Serum adiponectin	Per 1 log μg/ml increase	2623/11,986	14	RR	0.72 (0.67–0.78)	4.51 × 10^−16^	0.59–0.89	42.4	No/Yes	Highly suggestive
Sabanayagam, 2015 [65]	Central retinal arteriolar equivalent	Per 20 μm decrease	2581/16,190	5	HR	0.95 (0.86–1.06)	0.369	0.68–1.33	61.6	No/No	Not significant
Sabanayagam, 2015 [65]	Central retinal venular retinal equivalent	Per 20 μm increase	2581/16,190	5	HR	1.08 (1.02–1.15)	7.80 × 10^−3^	0.93–1.26	30.7	Yes/No	Weak
Sing, 2015 [66]	Serum calcium	Highest vs. lowest category	1476/32,641	3	HR	1.40 (1.11–1.75)	4.19 × 10^−3^	0.19–10.08	24.6	Yes/No	Weak
Song, 2013 [44]	Serum vitamin D	Highest vs. lowest category	5142/71,115	21	RR	0.62 (0.54–0.70)	1.44 × 10^−13^	0.46–0.83	19.4	No/No	Convincing
Wang, 2013 [67]	Serum CRP	Per 1 log pm/ml increase	5750/35,097	22	RR	1.26 (1.16–1.37)	5.79 × 10^−8^	0.92–1.71	63.9	No/Yes	Highly suggestive
Wang, 2013 [67]	Serum IL-6	Per 1 log pm/ml increase	4480/15,229	11	RR	1.31 (1.17–1.46)	3.40 × 10^−6^	0.97–1.75	42.5	No/Yes	Suggestive
Wang, 2015 [68]	Resting heart rate	Highest vs. lowest category	10,049/169,329	9	HR	1.57 (1.29–1.92)	6.11 × 10^−6^	0.83–2.98	84.3	No/No	Suggestive
Wu, 2012 [69]	Serum EPA and DHA	Per 3% of total fatty acids increase	1581/8801	5	RR	0.94 (0.75–1.17)	0.566	0.50–1.76	40.1	No/No	Not significant
Wu, 2012 [69]	Serum ALA	Per 0.1% of total fatty acids increase	1833/11,458	6	RR	0.89 (0.79–1.01)	0.064	0.69–1.14	17.1	Yes/Yes	Not significant
Yarmolinsky, 2016 [70]	Serum PAI-1	Highest vs. lowest category	980/8276	8	OR	1.67 (1.28–2.18)	1.38 × 10^−4^	0.88–3.18	38.2	No/Yes	Weak
*Dietary factors*											
Afshin, 2014 [71]	Nuts consumption	Per 4 servings/week increase	13,308/216,908	6	RR	0.87 (0.81–0.93)	9.49 × 10^−5^	0.75–1.01	21.1	No/No	Suggestive
Alhazmi, 2012 [72]	Total protein intake	Highest vs. lowest category	6290/201,223	3	HR	1.02 (0.90–1.17)	0.733	0.35–2.99	19	No/No	Not significant
Aune, 2009 [23]	Processed meat consumption	Highest vs. lowest category	9999/370,607	9	RR	1.41 (1.25–1.59)	3.03 × 10^−8^	1.01–1.98	52.5	No/No	Highly suggestive
Aune, 2009 [23]	Processed meat consumption	Per 50 g/day increase	9,456/362,749	8	RR	1.57 (1.28–1.93)	1.85 × 10^−5^	0.84–2.94	74.1	No/No	Suggestive
Aune, 2009 [23]	Total meat consumption	Highest vs. lowest category	6525/438,798	5	RR	1.17 (0.92–1.48)	0.193	0.49–2.81	86.9	No/No	Not significant
Aune, 2009 [23]	Total meat consumption	Per 120 g/day increase	5579/174,626	4	RR	1.26 (0.84–1.88)	0.259	0.21–7.61	90.8	No/No	Not significant
Aune, 2009 [23]	Total red meat consumption	Highest vs. lowest category	12,226/420,844	10	RR	1.21 (1.07–1.38)	3.08 × 10^−3^	0.83–1.76	58.5	No/No	Weak
Aune, 2009 [23]	Total red meat consumption	Per 120 g/day increase	10,305/387,067	9	RR	1.20 (1.04–1.38)	0.014	0.76–1.87	68.4	No/No	Weak
Aune, 2013 [73]	Dairy products	Per 400g/day increase	21,996/319,537	12	RR	0.93 (0.87–0.99)	0.019	0.81–1.07	31.9	No/No	Weak
Aune, 2013 [73]	Dairy products	Highest vs. lowest category	26,966/399,089	14	RR	0.89 (0.82–0.96)	3.24 × 10^−3^	0.72–1.10	42.2	No/No	Weak
Aune, 2013 [26]	Refined grains	Highest vs. lowest category	9547/248,531	6	RR	0.94 (0.82–1.09)	0.444	0.61–1.47	63.8	Yes/No	Not significant
Aune, 2013 [26]	Refined grains	Per 3 servings/day increase	9547/248,531	6	RR	0.96 (0.88–1.04)	0.320	0.75–1.22	52.6	No/No	Not significant
Aune, 2013 [26]	Whole grains	Highest vs. lowest category	19,107/364,443	9	RR	0.74 (0.70–0.78)	5.45 × 10^−30^	0.70–0.79	0	No/No	Convincing
Aune, 2013 [26]	Whole grains	Per 3 servings/day increase	19,831/366,037	10	RR	0.68 (0.57–0.81)	1.47 × 10^−5^	0.38–1.24	82.5	No/Yes	Suggestive
Bhupathiraju, 2014 [74]	Glycemic index	Highest vs. lowest category	36,562/400,485	20	RR	1.12 (1.03–1.21)	8.98 × 10^−3^	0.82–1.52	68.5	No/No	Weak
Bhupathiraju, 2014 [74]	Glycemic load	Highest vs. lowest category	NA/NA	30	RR	1.12 (1.06–1.17)	3.07 × 10^−5^	0.96–1.29	26.4	No/NA	Suggestive
Bi, 2015 [75]	Breakfast skipping	Yes vs. no	7419/99,516	8	RR	1.15 (1.04–1.27)	6.35 × 10^−3^	0.90–1.47	50	No/No	Weak
de Souza, 2015 [76]	Total saturated fat	Highest vs. lowest category	8739/228,715	8	RR	0.95 (0.88–1.03)	0.206	0.87–1.05	0	No/No	Not significant
de Souza, 2015 [76]	Total saturated fatty acids	Highest vs. lowest category	9758/234,788	10	RR	1.00 (0.90–1.12)	0.945	0.76–1.33	41.6	No/No	Not significant
de Souza, 2015 [76]	Total trans fat	Highest vs. lowest category	8690/221,445	6	RR	1.10 (0.95–1.26)	0.216	0.70–1.71	66	No/No	Not significant
de Souza, 2015 [76]	Total trans unsaturated fat	Highest vs. lowest category	9923/227,734	9	RR	0.98 (0.82–1.18)	0.828	0.54–1.77	78.1	No/No	Not significant
de Souza, 2015 [76]	Trans palmitoleic acid	Highest vs. lowest category	1153/11,789	5	RR	0.58 (0.46–0.74)	1.09 × 10^−5^	0.31–1.08	30.8	No/No	Suggestive
Ding, 2014 [77]	Coffee consumption	Highest vs. lowest category	50,273/1,046,597	32	RR	0.70 (0.65–0.75)	1.52 × 10^−25^	0.54–0.90	50.3	No/No	Highly suggestive
Djousse, 2016 [78]	Eggs consumption	Highest vs. lowest category	8911/211,068	12	RR	1.06 (0.86–1.30)	0.610	0.53–2.10	73.6	No/No	Not significant
Dong, 2012 [79]	Dietary calcium intake	Highest vs. lowest category	11,195/253,023	7	RR	0.85 (0.75–0.97)	0.018	0.59–1.23	53.4	No/No	Weak
Esposito, 2014 [28]	Healthy dietary pattern	Highest vs. lowest category	15,574/350,610	18	RR	0.80 (0.76–0.84)	4.86 × 10^−17^	0.73–0.88	8.6	No/No	Convincing
Greenwood, 2013 [80]	Glycemic index	Per 5 units/day increase	16,419/422,326	15	RR	1.08 (1.02–1.14)	0.013	0.87–1.34	87.6	No/Yes	Weak
Greenwood, 2013 [80]	Glycemic load	Per 20 units/day increase	24,942/486,351	16	RR	1.03 (1.00–1.05)	0.034	0.96–1.10	52.7	No/Yes	Weak
Greenwood, 2013 [80]	Carbohydrates consumption	Per 50 g/day increase	11,976/285,117	8	RR	0.97 (0.90–1.06)	0.514	0.75–1.26	75.5	No/Yes	Not significant
Guo, 2015 [81]	Nuts consumption	Highest vs. lowest category	11,580/251,083	6	RR	0.98 (0.84–1.15)	0.827	0.61–1.58	67.7	No/Yes	Not significant
Hu, 2012 [82]	Rice consumption	Highest vs. lowest category	13,583/338,765	7	RR	1.27 (1.04–1.54)	0.020	0.67–2.38	72	No/No	Weak
Imamura, 2015 [24]	Artificially-sweetened beverages	Per 1 serving/day increase	29,448/263,765	9	RR	1.07 (1.03–1.10)	1.32 × 10^−4^	0.99–1.14	28.8	No/Yes	Suggestive
Imamura, 2015 [24]	Fruit juice consumption	Per 1 serving/day increase	33,172/363,805	12	RR	1.07 (1.01–1.14)	0.031	0.90–1.27	50.9	No/No	Weak
Imamura, 2015 [24]	Sugar-sweetened beverages	Per 1 serving/day increase	38,253/426,684	17	RR	1.12 (1.06–1.20)	2.47 × 10^−4^	0.90–1.40	77.2	No/Yes	Suggestive
InterAct consortium, 2015 [83]	Total dietary fiber intake	Per 10 g/day increase	57,407/326,028	15	RR	0.91 (0.87–0.96)	3.43 × 10^−4^	0.81–1.03	31	No/No	Suggestive
Koloverou, 2014 [84]	Mediterranean diet	Highest vs. lowest category	19,663/115,923	10	RR	0.83 (0.74–0.93)	2.03 × 10^−3^	0.60–1.15	59	No/No	Weak
Kunutsor, 2013 [25]	Dietary heme iron	Highest vs. lowest category	7708/151,415	3	RR	1.28 (1.16–1.41)	3.35 × 10^−7^	0.69–2.37	0	No/No	Highly suggestive
Larsson, 2007 [85]	Magnesium intake	Per 100 mg/day increase	10,912/275,988	8	RR	0.85 (0.79–0.92)	1.43 × 10^−5^	0.69–1.06	65.8	No/Yes	Suggestive
Leermakers, 2016 [86]	Lutein intake	Highest vs. lowest category	1661/33,581	5	RR	0.97 (0.77–1.22)	0.783	0.50–1.89	48.8	No/No	Not significant
Li, 2014 [87]	Vegetables consumption	Highest vs. lowest category	20,933/269,994	9	RR	0.90 (0.80–1.01)	0.068	0.64–1.27	66.5	No/No	Not significant
Li, 2016 [88]	Alcohol consumption	Moderate drinkers vs. never drinkers	30,436/647,388	25	RR	0.74 (0.67–0.82)	4.86 × 10^−9^	0.49–1.10	74.4	No/No	Highly suggestive
Liu, 2014 [89]	Flavonoids intake	Highest vs. lowest category	18,146/266,460	6	RR	0.92 (0.87–0.98)	6.68 × 10^−3^	0.81–1.05	25.8	No/No	Weak
Tajima, 2014 [90]	Cholesterol intake	Highest vs. lowest category	7589/196,314	6	RR	1.24 (1.10–1.40)	4.93 × 10^−4^	0.91–1.68	41.4	No/No	Suggestive
Tajima, 2014 [90]	Cholesterol intake	Per 100 mg/day increase	6268/155,131	5	RR	1.09 (1.03–1.16)	4.34 × 10^−3^	0.91–1.31	50.4	No/No	Weak
Wang, 2015 [91]	Fruit consumption	Highest vs. lowest category	33,987/474,591	13	RR	0.92 (0.87–0.97)	1.92 × 10^−3^	0.83–1.01	11.2	No/No	Weak
Wang, 2015 [92]	Sugar-sweetened beverages	Highest vs. lowest category	30,005/347,941	9	RR	1.30 (1.21–1.41)	2.31 × 10^−12^	1.14–1.49	12.6	No/No	Convincing
Wu, 2012 [69]	Dietary ALA	Per 0.5 g/day increase	7365/124,575	7	RR	0.93 (0.83–1.04)	0.177	0.69–1.24	53	No/No	Not significant
Wu, 2012 [69]	Dietary EPA and DHA	Per 250 mg/day increase	23,739/500,199	16	RR	1.04 (0.97–1.10)	0.274	0.82–1.31	81.3	No/Yes	Not significant
Wu, 2012 [69]	Fish and seafood consumption	Per 100 g/day increase	20,830/460,659	13	RR	1.12 (0.94–1.34)	0.203	0.60–2.10	82.7	No/No	Not significant
Xi, 2014 [93]	Fruit juice	Highest vs. lowest category	19,986/355,275	8	RR	1.14 (1.03–1.27)	0.010	0.89–1.47	43.5	No/No	Weak
Yang, 2014 [94]	Tea consumption	Highest vs. lowest category	15,488/364,344	12	RR	0.84 (0.73–0.97)	0.014	0.57–1.23	42.5	Yes/No	Weak
Yao, 2014 [95]	Total dietary fiber intake	Highest vs. lowest category	14,973/355,422	12	HR	0.81 (0.73–0.90)	1.04 × 10^−4^	0.60–1.09	53.6	No/Yes	Suggestive
Zhao, 2014 [96]	Vitamin D intake	Highest vs. lowest category	9456/178,096	5	RR	0.93 (0.85–1.01)	0.067	0.81–1.06	0	No/No	Not significant
*Lifestyle and environmental factors*											
Aune, 2015 [97]	Leisure time physical activity	Highest vs. lowest category	151,523/1,669,717	55	RR	0.75 (0.70–0.79)	4.71 × 10^−22^	0.54–1.03	84	Yes/Yes	Highly suggestive
Aune, 2015 [97]	Leisure time physical activity	Per 5 hours/week increase	63,049/891,089	10	RR	0.75 (0.66–0.85)	4.44 × 10^−6^	0.51–1.11	90	Yes/Yes	Suggestive
Aune, 2015 [97]	Total physical activity	Highest vs. lowest category	17,103/87,459	14	RR	0.65 (0.59–0.71)	2.87 × 10^−21^	0.54–0.78	18.4	Yes/No	Highly suggestive
Biswas, 2015 [98]	Sedentary time	Highest vs. lowest category	6712/157,247	5	HR	1.91 (1.66–2.19)	9.30 × 10^−20^	1.52–2.39	0	No/No	Convincing
Capuccio, 2010 [99]	Difficulty in initiating sleep	Yes vs. no	787/23,405	6	RR	1.57 (1.26–1.97)	8.54 × 10^−5^	1.14–2.17	0	No/No	Weak
Capuccio, 2010 [99]	Difficulty in maintaining sleep	Yes vs. no	544/17,669	6	RR	1.84 (1.39–2.43)	2.16 × 10^−5^	1.00–3.37	22.3	No/No	Weak
Capuccio, 2010 [99]	Sleep duration	Long vs. normal	2903/85,708	7	RR	1.48 (1.12–1.96)	5.48 × 10^−3^	0.77–2.84	37.9	No/No	Weak
Galling, 2016 [100]	Antipsychotics	Yes vs. no	796/530,315	8	RR	3.02 (1.70–5.35)	1.56 × 10^−4^	0.46–19.63	89.8	No/No	Weak
Grontved, 2011 [101]	Television watching	Per 2 hours/day increase	6428/169,510	4	RR	1.20 (1.14–1.27)	5.66 × 10^−11^	0.98–1.47	50.3	No/No	Highly suggestive
Holliday, 2013 [102]	Sleep duration	Short vs. normal	17,660/429,464	12	OR	1.38 (1.18–1.60)	3.23 × 10^−5^	0.96–1.97	33.2	No/No	Suggestive
Leong, 2014 [103]	Spousal diabetes	Yes vs. no	5689/69,809	4	OR	1.39 (1.04–1.87)	0.026	0.44–4.47	59.6	Yes/No	Weak
Pan, 2015 [52]	Passive smoking	Ever vs. never	7843/148,596	7	RR	1.22 (1.10–1.35)	1.21 × 10^−4^	0.97–1.54	31.8	No/Yes	Suggestive
Pan, 2015 [52]	Smoking	Former vs. never smokers	161,938/2,714,859	47	RR	1.14 (1.10–1.19)	5.97 × 10^−12^	0.98–1.34	64	Yes/No	Highly suggestive
Pan, 2015 [52]	Smoking	Current vs. never smokers	270,705/5,580,157	88	RR	1.39 (1.33–1.44)	6.10 × 10^−65^	1.10–1.74	70.2	Yes/Yes	Highly suggestive
Pan, 2015 [52]	Smoking cessation	New quitters vs. never smokers	49,457/1,046,789	13	RR	1.54 (1.36–1.75)	2.13 × 10^−11^	0.99–2.40	82.5	Yes/Yes	Highly suggestive
Pan, 2015 [52]	Smoking cessation	Middle-term quitters vs. never smokers	39,130/1,033,615	11	RR	1.18 (1.07–1.29)	5.24 × 10^−4^	0.92–1.50	55.8	No/No	Suggestive
Pan, 2015 [52]	Smoking cessation	Long-term quitters vs. never smokers	48,357/988,055	11	RR	1.11 (1.02–1.21)	0.014	0.85–1.44	76.3	Yes/No	Weak
Wang, 2014 [104]	NO_2_	Per 10 μg/m^3^ increase	5113/69,922	6	RR	1.11 (1.07–1.16)	6.44 × 10^−7^	1.00–1.24	46.1	No/Yes	Highly suggestive
Wang, 2014 [104]	PM_10_	Per 10 μg/m^3^ increase	4974/92,653	4	RR	1.34 (1.22–1.47)	4.26 × 10^−10^	1.10–1.65	0	No/No	Convincing
Wang, 2014 [104]	PM_2.5_	Per 10 μg/m^3^ increase	16,165/2,284,699	5	RR	1.39 (1.14–1.68)	8.18 × 10^−4^	0.73–2.63	86.3	No/No	Suggestive
Wu, 2013 [105]	Persistent organic pollutants	Highest vs. lowest category	381/3672	8	OR	1.70 (1.23–2.35)	1.24 × 10^−3^	0.93–3.13	16	No/No	Weak
Zaccardi, 2015 [106]	Cardiorespiratory fitness	Per 1 metabolic equivalent increase	8564/84,428	8	HR	0.95 (0.92–0.98)	2.98 × 10^−3^	0.86–1.05	88.1	No/Yes	Weak
*Medical history*											
Aune, 2014 [107]	Breastfeeding*	Highest vs. lowest category	10,842/263,119	6	RR	0.68 (0.57–0.82)	3.75 × 10^−5^	0.38–1.22	74.7	No/Yes	Suggestive
Aune, 2014 [107]	Breastfeeding*	Per 12 months increase	10,306/261,523	4	RR	0.91 (0.86–0.96)	7.24 × 10^−4^	0.72–1.16	81.1	No/Yes	Suggestive
Bellamy, 2009 [37]	Gestational diabetes	Yes vs. no	10,859/664,596	20	RR	7.43 (4.79–11.51)	3.09 × 10^−19^	1.57–35.07	85.9	No/No	Highly suggestive
Coto, 2013 [108]	Psoriasis	Yes vs. no	255,203/5,393,406	38	OR	1.69 (1.50–1.89)	1.60 × 10^−19^	0.88–3.24	98.1	No/No	Highly suggestive
Coto, 2013 [108]	Psoriatic arthritis	Yes vs. no	1420/15,494	3	OR	2.18 (1.36–3.48)	1.20 × 10^−3^	0.01–395.32	77.2	Yes/No	Weak
Ford, 2008 [38]	Metabolic syndrome	Yes vs. no	2248/29,401	14	HR	3.35 (2.75–4.08)	4.69 × 10^−33^	1.66–6.74	74.6	Yes/No	Highly suggestive
Horta, 2015 [109]	Breastfeeding**	Ever vs. never	NA/NA	11	OR	0.65 (0.49–0.86)	2.66 × 10^−3^	0.31–1.37	52.6	No/NA	Weak
Janghorbani, 2014 [110]	Age at menarche	Highest vs. lowest category	21,095/294,333	12	RR	1.25 (1.15–1.35)	5.77 × 10^−8^	0.99–1.58	66.6	No/No	Highly suggestive
Li, 2014 [111]	Preterm birth	Preterm vs. normal term	1898/29,580	5	RR	1.51 (1.33–1.72)	4.54 × 10^−10^	1.22–1.87	0	No/No	Convincing
Louati, 2015 [112]	Osteoarthritis	Yes vs. no	130,457/909,718	20	OR	1.41 (1.21–1.65)	1.36 × 10^−5^	0.81–2.47	95.2	No/No	Suggestive
Moran, 2010 [113]	PCOS	Yes vs. no	2337/66,727	13	OR	3.14 (1.86–5.31)	1.80 × 10^−5^	0.86–11.49	55.5	No/No	Suggestive
Stubbs, 2015 [114]	Schizophrenia	Yes vs. no	131,675/2,147,884	26	OR	1.83 (1.53–2.18)	2.63 × 10^−11^	0.79–4.20	98.1	Yes/Yes	Suggestive
Ungprasert, 2015 [115]	Giant cell arteritis	Yes vs. no	284/1683	5	OR	0.74 (0.57–0.96)	0.025	0.49–1.13	0	No/No	Weak
Vancampfort, 2015 [116]	Major depressive disorder	Yes vs. no	128,807/2,123,622	10	OR	1.48 (1.28–1.71)	8.11 × 10^−8^	0.95–2.33	87.2	No/No	Highly suggestive
Vancampfort, 2015 [117]	Bipolar disorder	Yes vs. no	87,168/702,464	5	OR	1.98 (1.62–2.41)	1.14 × 10^−11^	1.01–3.86	76.8	No/No	Highly suggestive
Wang, 2013 [118]	Obstructive sleep apnea	Yes vs. no	422/5940	6	RR	1.63 (1.09–2.45)	0.018	0.60–4.48	41.2	No/Yes	Weak
*Metabolic and anthropometric traits*											
Abdullah, 2010 [119]	BMI	Obese vs. lean	16,109/574,142	18	RR	6.88 (5.39–8.78)	4.20 × 10^−54^	2.39–19.81	91.1	No/No	Highly suggestive
Abdullah, 2010 [119]	BMI	Overweight vs. lean	15,796/419,466	17	RR	2.93 (2.33–3.68)	2.80 × 10^−20^	1.11–7.76	90.6	No/No	Highly suggestive
Bell, 2014 [120]	Metabolically healthy obesity	Metabolically healthy obese vs. metabolically healthy non-obese	1285/26,196	10	RR	4.40 (2.83–6.84)	4.97 × 10^−11^	1.29–14.95	47.8	No/No	Convincing
Bell, 2014 [120]	Metabolically healthy obesity	Metabolically unhealthy obese vs. metabolically healthy non-obese	1266/24,668	8	RR	9.50 (7.48–12.08)	8.79 × 10^−76^	7.05–12.82	0	Yes/No	Highly suggestive
Harder, 2007 [121]	Birth weight	>4,000 g vs. <4,000 g	6005/108,400	9	OR	1.27 (1.01–1.59)	0.044	0.62–2.58	68.2	No/No	Weak
Harder, 2007 [121]	Birth weight	>2,500g vs. <2,500g	5815/100,759	10	OR	1.32 (1.06–1.64)	0.014	0.71–2.43	60.8	No/No	Weak
Janghorbani, 2012 [122]	Height	Highest vs. lowest category	2858/66,199	17	OR	0.85 (0.76–0.96)	6.65 × 10^−3^	0.58–1.25	61.3	Yes/Yes	Weak
Janghorbani, 2012 [122]	Hip circumference	Highest vs. lowest category	5415/169,924	18	OR	0.57 (0.48–0.68)	6.72 × 10^−10^	0.32–1.05	62.9	No/No	Highly suggestive
Kodama, 2012 [123]	BMI	Per 1 SD increase	10,043/132,442	15	RR	1.59 (1.40–1.80)	3.99 × 10^−13^	0.95–2.65	94.3	No/Yes	Highly suggestive
Kodama, 2012 [123]	Waist circumference	Per 1 SD increase	10,043/132,442	15	RR	1.66 (1.47–1.88)	1.14 × 10^−15^	1.00–2.76	94.5	No/Yes	Highly suggestive
Kodama, 2012 [123]	Waist-height ratio	Per 1 SD increase	10,043/132,442	15	RR	1.67 (1.46–1.90)	3.68 × 10^−14^	0.97–2.87	94.2	No/Yes	Highly suggestive
Kodama, 2012 [123]	Waist-to-hip ratio	Per 1 SD increase	10,043/132,442	15	RR	1.54 (1.36–1.75)	1.86 × 10^−11^	0.93–2.56	93.7	No/Yes	Highly suggestive
Kodama, 2014 [124]	Weight gain in early adulthood	Per 5 kg/m^2^ increase	15,701/327,002	10	RR	3.07 (2.49–3.80)	1.92 × 10^−25^	1.39–6.78	98.2	No/No	Highly suggestive
Kodama, 2014 [124]	Weight gain after the age of 25 years	Per 5 kg/m^2^ increase	13,364/294,135	15	RR	2.12 (1.74–2.58)	5.03 × 10^−14^	1.07–4.20	75.1	Yes/No	Highly suggestive
Whincup, 2008 [125]	Birth weight	Per 1 kg increase	6090/145,994	31	OR	0.80 (0.72–0.88)	1.84 × 10^−5^	0.52–1.21	66.5	No/No	Suggestive
Agardh, 2011 [36]	Educational status	Lowest vs. highest category	20,649/234,796	23	RR	1.41 (1.28–1.55)	1.01 × 10^−12^	1.02–1.96	65.5	Yes/No	Highly suggestive
Agardh, 2011 [36]	Income level	Lowest vs. highest category	1837/19,049	7	RR	1.40 (1.04–1.88)	0.029	0.56–3.47	72	Yes/Yes	Weak
Agardh, 2011 [36]	Occupation	Lowest vs. highest category	2691/42,476	11	RR	1.31 (1.09–1.57)	3.69 × 10^−3^	0.77–2.21	52.7	No/No	Weak
Huang, 2015 [126]	Adverse childhood experience	Yes vs. no	3481/83,770	7	OR	1.28 (1.05–1.55)	0.014	0.76–2.16	60.9	No/No	Weak
Jokela, 2014 [35]	Agreeableness	Per 1 SD increase in personality score	1845/33,058	5	OR	1.05 (0.98–1.13)	0.193	0.85–1.30	40.6	No/No	Not significant
Jokela, 2014 [35]	Conscientiousness	Per 1 SD increase in personality score	1845/33,058	5	OR	0.86 (0.82–0.91)	9.94 × 10^−8^	0.79–0.94	0	No/No	Convincing
Jokela, 2014 [35]	Extraversion	Per 1 SD increase in personality score	1845/33,058	5	OR	1.01 (0.94–1.09)	0.742	0.84–1.22	32.5	No/No	Not significant
Jokela, 2014 [35]	Neuroticism	Per 1 SD increase in personality score	1845/33,058	5	OR	1.06 (1.00–1.13)	0.062	0.91–1.24	26.7	No/No	Not significant
Jokela, 2014 [35]	Openness	Per 1 SD increase in personality score	1845/33,058	5	OR	0.96 (0.85–1.08)	0.453	0.62–1.46	77.7	No/No	Not significant
Kivimaki, 2015 [127]	Working hours	Long vs. standard working hours	4963/217,157	23	RR	1.09 (0.91–1.30)	0.366	0.58–2.04	53.3	No/No	Not significant
Nyberg, 2014 [128]	Job strain	Highest vs. lowest category	3703/121,105	13	HR	1.15 (1.06–1.25)	1.46 × 10^−3^	1.04–1.27	0	No/No	Weak

γGT: gamma-glutamyl transferase, ALA: α-linolenic acid, ALT: alanine aminotransferase, AST: aspartate aminotransferase, BMI: body mass index, CI: confidence interval, CRP: C-reactive protein, DHA: docosahexaenoic acid, EPA: eicosapentaenoic acid, HR: hazard ratio, IL-6: interleukin-6, NA: not available, NO_2_: nitrogen dioxide, OR: odds ratio, PAI-1: plasminogen activator inhibitor-1, PCOS: polycystic ovary syndrome, PM_2,5_: particulate matter with a diameter of 2,5 μm or less, PM_10_: particulate matter with a diameter between 2,5 and 10 μm, RR: risk ratio, SD: standard error

*maternal risk for T2DM

**offspring risk for T2DM

Thirty-eight associations (27%) were very heterogeneous (I^2^ >75%), and 50 associations (35%) had large heterogeneity estimates (I^2^ ≥50% and I^2^ ≤75%). The Egger’s test was statistically significant in 32 meta-analyses (23%), and 27 of them presented evidence for small-study effects. Thirty-nine meta-analyses (28%) had evidence for excess significance bias.

### Assessment of epidemiological credibility

Eleven associations (8%) presented convincing evidence (>1,000 cases, P <10^−6^, not large between-study heterogeneity, 95% PI excluding the null value, no evidence for small-study effects and excess significance bias) for risk of T2DM. Low whole grains consumption, metabolically healthy obesity, increased sedentary time, low adherence to a healthy dietary pattern, high level of serum uric acid, low level of serum vitamin D, decreased conscientiousness, preterm birth, high consumption of sugar-sweetened beverages, high level of serum ALT, and exposure to high level of PM_10_ were associated with increased risk for T2DM and supported by convincing evidence.

Thirty-four associations (24%) were supported by highly suggestive evidence. The associations that were linked with a higher risk for T2DM and presented highly suggestive evidence were the following: high BMI (obese vs. lean, overweight vs. lean, and per 1 SD increase), low educational status, gestational diabetes, increased processed meat consumption, high level of total and leisure-time physical activity, metabolically unhealthy obesity, psoriasis, low coffee consumption, high systolic blood pressure, high level of serum gamma-glutamyl transferase (highest vs. lowest category, and per 1 log unit increase), metabolic syndrome, increased time of television watching, low hip circumference, late age at menarche, weight gain in early adulthood, weight gain after the age of 25 years, increased dietary heme iron intake, high level of serum C-reactive protein (highest vs. lowest category, and per 1 log pm/mL), low level of serum adiponectin (per 1 log μg/ml increase), low alcohol consumption, smoking (former vs. never smokers, and current vs. never smokers), smoking cessation (new quitters vs. never smokers), major depressive disorder, bipolar disorder, high waist-height ratio, high waist circumference, high waist-to-hip ratio, and exposure to high level of NO_2_ (per 10 μg/m^3^ increase). Twenty-nine associations had suggestive evidence (20%), and 42 associations had weak evidence (30%) for risk of T2DM.

**Fig 3:**
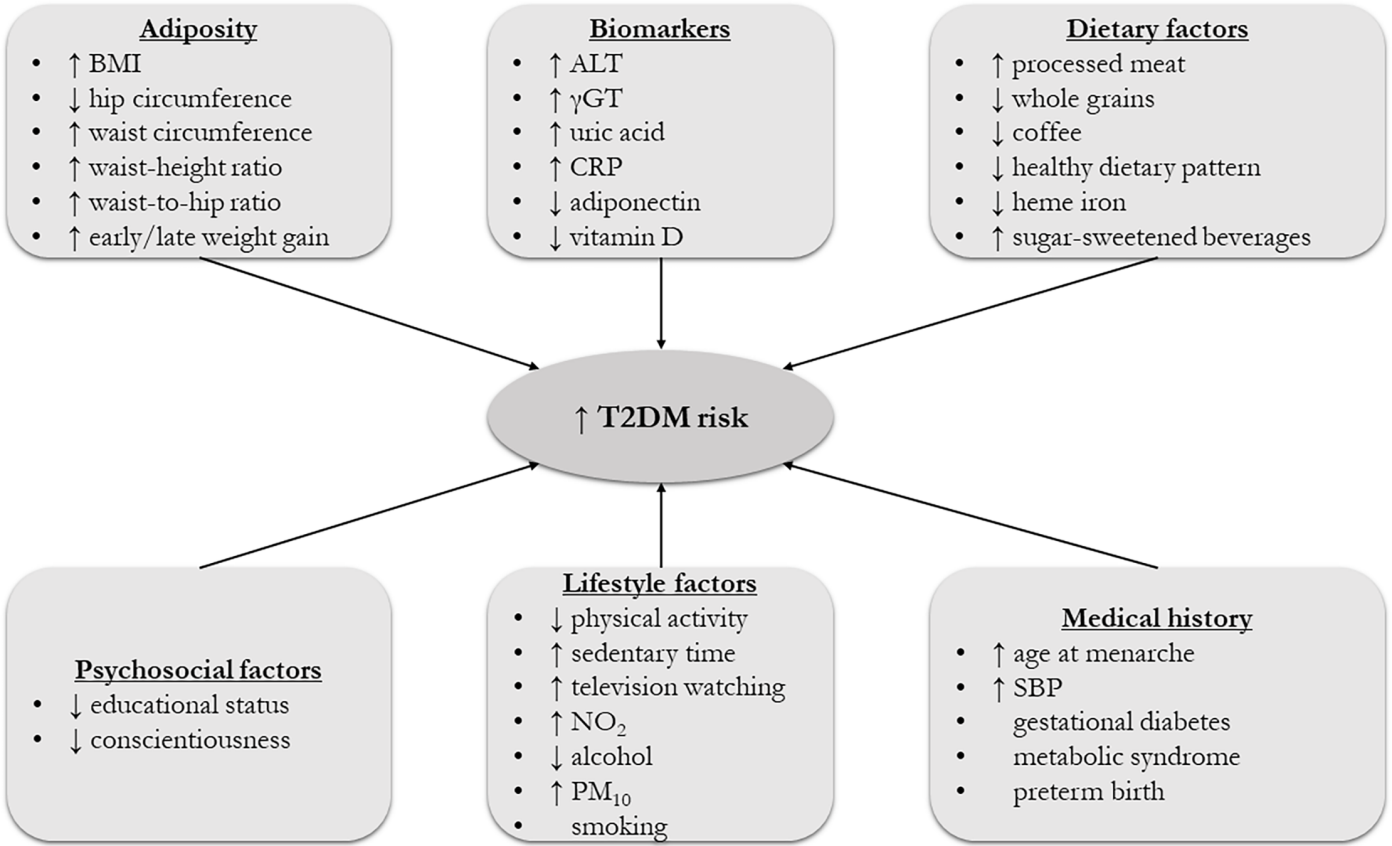
Schematic representation of risk factors for T2DM with convincing or highly suggestive evidence. The symbol ↑ denotes a higher exposure to a risk factor, and the symbol ↓ represents a lower exposure to a risk factor. For alcohol consumption, never drinkers presented a higher risk for T2DM than moderate drinkers.

**Fig 4:**
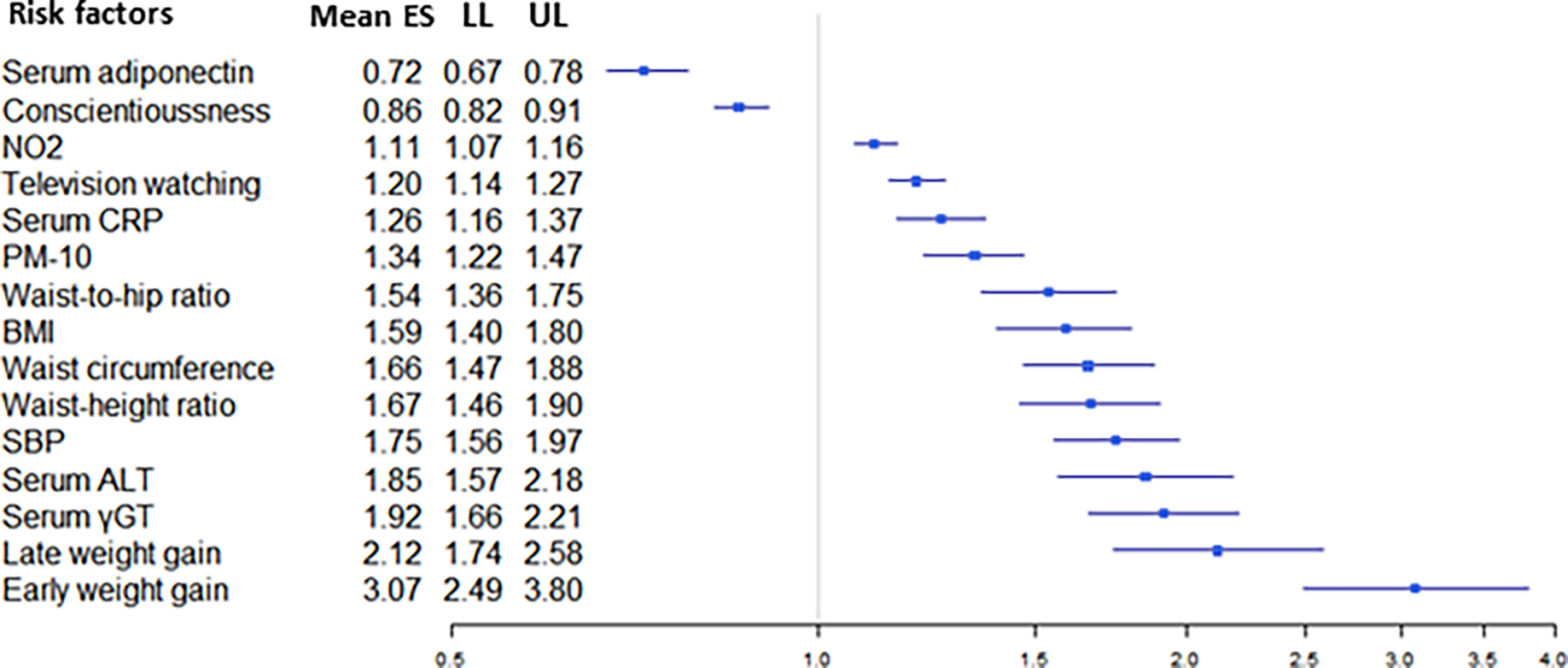
Forest plot of risk factors (measured as continuous variables) for T2DM supported by convincing or highly suggestive evidence.

**Fig 5:**
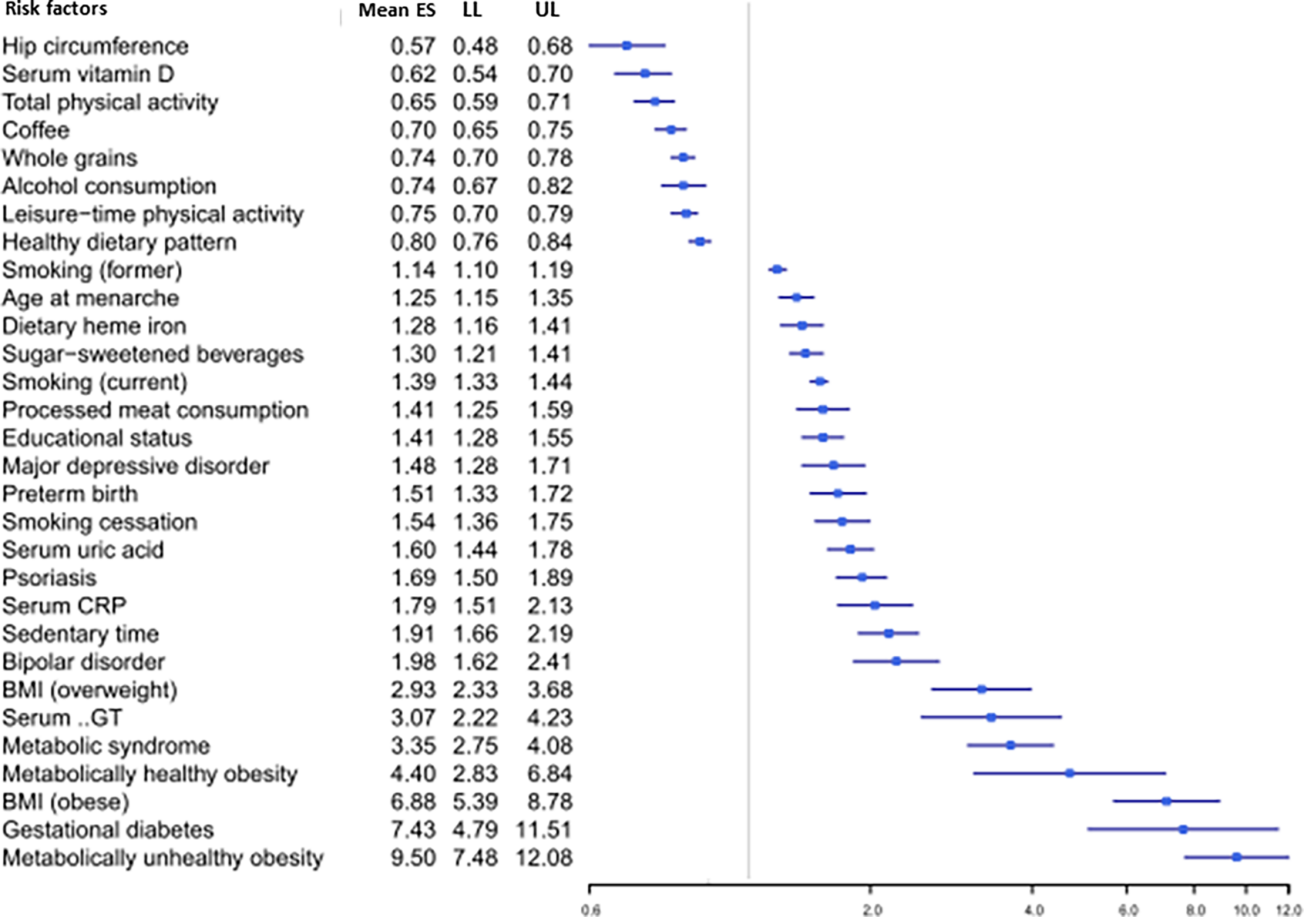
Forest plot of risk factors (measured as dichotomous variables) for T2DM supported by convincing or highly suggestive evidence.

All but 6 associations with convincing or highly suggestive evidence were exclusively based on prospective cohort studies, case-cohort studies and/or nested case-control studies. The remaining six associations (i.e., sedentary time, psoriasis, hip circumference, age at menarche, bipolar disorder, and major depressive disorder) were based on a combination of cross-sectional studies and cohort studies. In a sensitivity analysis limited to prospective cohort studies, the associations for sedentary time, hip circumference, and age at menarche remained highly suggestive (Table 2). For psoriasis, the level of evidence became suggestive due to a P-value greater than 10^−6^ under random-effects model (Table 2). In the meta-analysis for bipolar disorder, no prospective cohort studies were included. In the meta-analysis for major depressive disorder, only 1 retrospective cohort study was included. All the risk factors with convincing and highly suggestive evidence are summarized in Fig 3, and they are graphically presented using forest plots in Figs 4 and 5.

**Table 2:** Sensitivity analysis of prospective cohort studies for associations with convincing or highly suggestive evidence that were based on a combination of cross-sectional and cohort studies.

Reference	Risk factor	Level of comparison	Number of datasets	Number of cases/controls	Effect size metric	Random-effects summary effect size (95% CI)	P random	95% prediction interval	I^2^
Biswas, 2015 [98]	Sedentary time	Highest vs. lowest category	4	6428/151,290	HR	1.88 (1.63–2.17)	1.52 × 10^−17^	1.37–2.58	0
Coto, 2013 [108]	Psoriasis	Yes vs. no	8	49,064/1,564,468	OR	1.53 (1.29–1.81)	1.15 × 10^−6^	0.83–2.80	96.7
Janghorbani, 2012 [122]	Hip circumference	Highest vs. lowest category	11	4460/137,666	OR	0.63 (0.53–0.75)	3.76 × 10^−7^	0.39–1.01	50.4
Janghorbani, 2014 [110]	Age at menarche	Highest vs. lowest category	9	20,092/289,532	RR	1.26 (1.15–1.38)	5.44 × 10^−7^	0.96–1.64	72.7

CI: confidence interval, HR: hazard ratio, OR: odds ratio, RR: risk ratio

### Mendelian randomization studies

We identified 22 MR studies assessing the causal effect of a risk factor that was included in our umbrella review (Table 3). The median number of T2DM cases was 4,407 (IQR, 1,164–15,255). Two MR studies used a single SNP as instrumental variable and twenty MR studies constructed a polygenic risk score (PRS). In studies with PRS, the median number of variants was 5 (IQR, 3–8). The eligible MR studies assessed the following 13 exposures: alcohol intake, birth weight, BMI, coffee intake, milk intake, systolic blood pressure, serum adiponectin, serum CRP, serum ferritin, serum gamma-glutamyl transferase, serum uric acid, serum vitamin D, and waist circumference. Seven risk factors were examined by more than one MR study.

**Table 3:** Characteristics of mendelian randomization studies for type 2 diabetes mellitus.

Reference	Exposure	Level of comparison	Genetic instrument	N of SNPs in instrument	N cases	Effect size metric	Causal effect size (95% CI)	P-value
Holmes, 2014 [29]	Alcohol intake	Per units/week increase	Single variant (rs1229984)	1	14,549	OR	1.02 (0.95–1.09)	0.627
Wang, 2016 [54]	Birth weight	Per 1 SD decrease	PRS	5	3627	OR	2.94 (1.70–5.16)	<0.001
Afzal, 2014 [31]	BMI	Per 10 kg/m^2^ increase	PRS	3	5037	HR	19.40 (6.40–59.10)	NR
Corbin, 2016 [32]	BMI	Per 1 kg/m^2^ increase	PRS	96	12,171	OR	1.39 (1.14–1.68)	0.002
Fall, 2013 [33]	BMI	Per 1 kg/m^2^ increase	Single variant (rs9939609)	1	1991	OR	1.35 (1.12–1.62)	0.001
Holmes, 2014 [34]	BMI	Per 1 kg/m^2^ increase	PRS	14	4407	OR	1.27 (1.18–1.36)	2.0 × 10^−11^
Nordestgaard, 2015 [129]	Coffee intake	Per 1 cup/day	PRS	5	26,632	OR	1.00 (0.99–1.01)	NR
Bergholdt, 2015 [130]	Milk intake	Per 1 glass/week increase	Single variant (rs4988235)	1	951	OR	0.99 (0.93–1.06)	NR
Aikens, 2016 [131]	SBP	Per 1 mmHg increase	PRS	13	37,293	OR	1.02 (1.01–1.03)	9.1 × 10^−5^
Marott, 2016 [132]	SBP	Per 1 mmHg increase	PRS	6	2859	OR	0.97 (0.95–1.00)	0.030
Peters, 2013 [50]	Serum adiponectin	Per 1 SD decrease	PRS	3	967	OR	0.86 (0.75–0.99)	0.013
Yaghootkar, 2013 [51]	Serum adiponectin	Per 1 SD decrease	PRS	3	2777	OR	0.94 (0.75–1.19)	0.610
Yaghootkar, 2013 [51]	Serum adiponectin	Per 1 SD decrease	PRS	3	15,960	OR	0.99 (0.95–1.04)	0.770
Prins, 2016 [40]	Serum CRP	Per 10-s% increase	PRS	4	6698	OR	1.11 (0.94–1.32)	0.230
Prins, 2016 [40]	Serum CRP	Per 10-s% increase	PRS	18	6698	OR	1.09 (0.95–1.24)	0.210
Gan, 2012 [133]	Serum ferritin	Per 1 ng/mL increase	Single variant (rs855791)	1	272	OR	0.80 (0.65–0.98)	0.031
Gan, 2012 [133]	Serum ferritin	Per 1 ng/mL increase	Single variant (rs4820268)	1	272	OR	0.80 (0.66–0.98)	0.031
Lee, 2016 [134]	Serum gamma-glutamyl transferase	Per 1 unit increase	PRS	7	343	OR	1.05 (1.01–1.08)	NR
Kleber, 2015 [41]	Serum uric acid	Per 1 mg/dl increase	PRS	8	1236	OR	0.83 (0.57–1.23)	0.360
Pfister, 2011 [42]	Serum uric acid	Per 1 mg/dl increase	PRS	8	7504	OR	0.99 (0.94–1.04)	0.620
Slujis, 2015 [43]	Serum uric acid	Per 1 mg/dl increase	PRS	24	41,508	HR	0.99 (0.92–1.06)	NR
Afzal, 2014 [31]	Serum vitamin D	Per 20 nmol/L decrease	PRS	2	5037	HR	1.51 (0.98–2.33)	0.240
Afzal, 2014 [31]	Serum vitamin D	Per 20 nmol/L decrease	PRS	2	5037	HR	1.02 (0.75–1.37)	0.390
Buijsse, 2013 [45]	Serum vitamin D	Per 5 nmol/L increase	PRS	4	1572	HR	0.98 (0.89–1.08)	NR
Jorde, 2012 [46]	Serum vitamin D	Highest vs. lowest quartile	PRS	5	1092	HR	1.01 (0.86–1.20)	NR
Leong, 2014 [47]	Serum vitamin D	Per 1 SD increase	Single variant (rs2282679)	1	201	OR	0.99 (0.79–1.24)	0.930
Ye, 2015 [48]	Serum vitamin D	Per 1 SD increase	PRS	4	28,144	OR	1.01 (0.75–1.36)	0.940
Marott, 2016 [132]	Waist circumference	Per 1 unit increase	PRS	5	3762	OR	1.05 (1.01–1.10)	0.020

BMI: body mass index, CI: confidence interval, CRP: C-reactive protein, HR: hazard ratio, NR: not reported, OR: odds ratio, PRS: polygenic risk score, SBP: systolic blood pressure, SD: standard deviation. SNPs: single nucleotide polymorphisms

A causal effect was claimed for 4 risk factors graded as highly suggestive in our umbrella review: BMI, systolic blood pressure, serum gamma-glutamyl transferase, and waist circumference. A causal association was also claimed for birth weight, but a relatively small number of T2DM cases was included in this analysis. The observed effects for alcohol intake, coffee intake, serum CRP, serum ferritin, serum uric acid and serum vitamin D were not causal. Milk intake presented weak evidence in our analysis and an MR study did not show a causal effect. Serum adiponectin was graded as highly suggestive in our analysis, but the findings from MR studies were conflicting, and the largest MR study indicated absence of a causal effect.

## Discussion

We performed a mapping of environmental factors and biomarkers examined for an association with T2DM in systematic reviews and meta-analyses. Overall, more than 100 associations were considered. We identified eleven associations supported by convincing evidence and thirty-four additional associations having highly suggestive evidence for risk of T2DM. These associations mainly pertained to comorbid medical conditions, lifestyle and dietary factors, as well as serum biomarkers.

Even though more than one third of the associations examined various dietary factors, only six of them showed convincing or highly suggestive relationship with T2DM and the demonstrated effect sizes were modest. These factors were processed meat, whole grain products, healthy dietary pattern, sugar-sweetened beverages and dietary heme iron. Increased processed meat and sugar-sweetened beverages consumption are linked with other unhealthy lifestyle factors which showed highly significant association with T2DM, such as physical inactivity, increased BMI, smoking and unhealthy dietary patterns. [23,24] The association between dietary heme iron and T2DM could be explained by the fact that red meat is the main dietary source of heme iron. [25] The observed protective effect of whole grain products is independent of BMI as almost all observational studies have adjusted for its effect. Whole grain products have high concentration of fibers, which delay gastric emptying, therefore slowing glucose release in circulation. This results in reduced postprandial insulin response and could improve insulin sensitivity [26,27] The aforementioned associations are also supported by the observed protective effect of healthy dietary pattern against developing T2DM. Although the term “healthy dietary pattern” includes a variety of diets, the same principles apply: reduced red and processed meat consumption, moderate alcohol drinking, low intake of sugar-sweetened beverages and increased consumption of whole grain products. [28]

Moderate alcohol consumption has a protective effect against developing T2DM. This relationship could be explained by increased insulin sensitivity, lower fasting insulin resistance and lower glycated hemoglobin concentrations, which are induced by moderate amounts of alcohol. Moreover, moderate amount of alcohol drinking is a common feature of healthy diet pattern, who also lowered the risk for developing T2DM. Furthermore, coffee consumption lowers the risk for T2DM, which is attributed to the reduction of insulin resistance and the improvement of glucose metabolism. However, it is unclear whether this association is causal, given the findings of a recently published MR study [29].

Most of the associations yielded from our analyses were proxies of obesity and include body mass index (BMI), weight gain, and anthropometric characteristics (i.e., hip circumference, waist-height ratio, waist-hip ratio, waist circumference). The observed association between BMI and T2DM demonstrated a large effect size and was highly significant (RR = 6.88, P = 4.2 × 10^−54^). Increased BMI, waist-height ratio, waist-hip ratio and waist circumference express the presence of increased intra-abdominal visceral fat, which disrupts insulin metabolism through release of serum free fatty acids. [30] Not surprisingly, findings from MR studies further support a causal role of BMI in the pathogenesis of T2DM. [31–34] However, not all obese have the same risk for developing T2DM; it seems that the risk is affected by their metabolic profile. Metabolically unhealthy obese carry an about 10-fold risk for T2DM, whereas metabolically healthy obese have an about 4.5-fold risk for T2DM. Moreover, weight gain during early adulthood was more harmful than weight gain after the age of 25. On the contrary, peripheral fat accumulation has been linked to a better metabolic profile, which is depicted in the observed protective effect of larger hip circumference on T2DM.

Several lifestyle factors presented either convincing or highly suggestive evidence. Total and leisure-time physical activity lowered the relative risk for T2DM. High sedentary time and TV watching are inter-correlated, and they are surrogates of physical inactivity, which is a common feature in people with high BMI. Additionally, the convincing association of low conscientiousness with increased risk for T2DM could be explained by the correlation of this personality trait with physical inactivity and high risk for obesity. [35] Our analysis also indicated that there is highly suggestive evidence for the association of lower educational attainment and higher risk for T2DM. Educational level constitutes a component of socioeconomic status. Lower socioeconomic status is associated with higher stress levels, leading to disruption in endocrine function through perturbations in the neuroendocrine system. [36] Also, people with low socioeconomic status are more prone to an unhealthy lifestyle pattern and they have limited access to healthcare care facilities. [36]

Several medical conditions have been traditionally linked to increased risk for T2DM. Patients with metabolic syndrome and gestational diabetes presented higher risk for T2DM. The seven-fold increase of risk for developing T2DM in women with gestational diabetes could be attributed to common underlying genetic and environmental risk factors between the two conditions. [37] Metabolic syndrome is considered a predictor of T2DM and has a stronger association with T2DM than its components. [38] Furthermore, higher systolic blood pressure was associated with increased risk for T2DM, but this association might not be causal. Some antihypertensive drugs have been associated with an increased risk, whereas the use of antihypertensive drugs inhibiting the renin-angiotensin system showed a protective effect. In turn, increased activity of renin-angiotensin system induces systemic inflammation processes that may exert a diabetogenic effect. [39]

Our analysis showed that a set of serum biomarkers is highly associated with the risk for T2DM. These biomarkers pertained to serum level of alanine aminotransferase (ALT), gamma-glutamyl transferase, C-reactive protein (CRP), uric acid, adiponectin, and vitamin D. High serum ALT and gamma-glutamyl transferase in patients with T2DM could be a manifestation of ongoing low-grade hepatic inflammation or hepatocellular damage, which is common in T2DM and metabolic syndrome. Among hepatic enzymes, ALT is the most specific indicator of hepatic pathology in non-alcoholic fatty liver disease and most closely related to liver fat accumulation. The presence of systemic inflammation is linked to β-cell dysfunction, leading to impaired glucose metabolism and the development of T2DM. [4] Both CRP and uric acid are inflammatory markers associated with systemic inflammation. Also, meat consumption is directly associated with serum uric acid level, and, as we have already shown, processed meat consumption is linked to higher risk for T2DM. However, MR studies for serum CRP [40], and serum uric acid [41–43] suggested that the associations with T2DM might not be causal.

Furthermore, our results indicated an inverse association between vitamin D level and risk for T2DM. It is unclear if this is a true association or the effect of adiposity as a potential confounder or intermediate factor. Obesity leads to storage of vitamin D in adipose tissue and to less sun exposure, on the grounds of limited mobility and accumulation of subcutaneous fat [44]. All the former result in low circulating level of vitamin D in obese individuals. Also, vitamin D may directly affect adiposity and other metabolic parameters, such as dyslipidemia, hypertension, and systemic inflammation, that mediate the pathway from vitamin D status to T2DM. Adiponectin is another serum biomarker that expresses the body composition. It affects the glucose metabolism, and higher serum level of adiponectin are associated with higher insulin sensitivity. However, MR studies examining the role of serum vitamin D indicated a non-causal association that might be explained by confounding factors [31,45–48], whereas the evidence on the causal role of serum adiponectin are contradictory [49–51].

The association between smoking and T2DM has biological foundation because smoking is associated with central obesity and increased oxidative stress and inflammation, and eventually leads to insulin resistance and hyperglycaemia. However, residual confounding can be the case since smoking is often linked to other unhealthy lifestyle factors (e.g., poor diet, physical inactivity) and comorbidities. The increased risk of T2DM associated with smoking cessation in new quitters could be mediated by weight gain or be due to reverse causation because people who try to quit smoking are more likely to have preclinical conditions or high cumulative smoking exposure [52].

Based on our assessment, adults delivered preterm presented a larger risk for development of T2DM during adulthood than adults delivered full-term. According to the “fetal origin of disease” hypothesis, the biological mechanisms that mediate this association could be explained through intrauterine growth restriction. Preterm newborns have low birth weight and they are prone to disrupted glucose metabolism in later life [53], which in turn predisposes to an increased risk of T2DM. Although the association between birth weight and T2DM had weak epidemiological credibility [12], an MR study indicated that there is a potential causal association between birth weight and risk for T2DM [54].

Two components of ambient air pollution, PM_10_ and NO_2_, were found to have robust association with risk for T2DM. It has been suggested that air pollution causes elevated systemic inflammation and oxidative stress, whereas it increases the insulin resistance leading to abnormal glucose metabolism and elevated fasting glucose. [55]

Furthermore, older age at menarche was associated with risk for T2DM. However, there are doubts whether it constitutes a genuine association. Observational studies found that this association is attenuated after adjustment for BMI in adulthood, suggesting that adult adiposity may mediate this association. The inverse association between age at menarche and BMI in adulthood could explain this finding. [56]

We presented an exposure-wide mapping of the meta-analyses on non-genetic risk factors for T2DM. Our umbrella review indicated that a very wide range of risk factors has been considered for T2DM. Compared to previously published umbrella reviews [6–10], there is tremendous amount of meta-analyses for risk factors of T2DM. Also, the majority of these associations were examined in large prospective cohort studies. The increasing incidence and large burden of T2DM could explain the observed interest in the field of non-genetic and modifiable risk factors for T2DM.

Our study has some caveats. First, the statistical test for small-study effects should be interpreted with caution in case of large between-study heterogeneity. Second, the observational studies did not often clearly report the sample sizes for the statistical analyses. Thus, the power calculations might be conservative, and the extent of excess significance bias is probably conservative. Furthermore, genetic instruments of the MR studies were not assessed, and power calculations for the MR studies could not be performed, because the percentage of variance explained often was not available. Consequently, the claims of MR studies should be interpreted with caution.

## Conclusions

Our paper identified several robust risk factors for T2DM. Our findings indicate specific strategies for public health interventions to reduce the future incidence of T2DM. Interventions for the promotion of physical activity and a healthy lifestyle and dietary pattern combined with interventions against the increased incidence of obesity could alleviate the projections for an increase of T2DM incidence in near future. However, these findings are based on observational data and should be interpreted with caution. Even though MR studies may support or not causality, the power of those studies could not be assessed. Therefore, randomized clinical trials and additional well-designed MR studies are needed to clarify which of these observations are causal associations. Also, these findings should be replicated by large-scale environment-wide association studies in various ethnic groups, and they could be used for the development of reliable risk prediction models in combination with known genetic polymorphisms.

## Supporting information

S1 File PRISMA checklist. (DOC)
